# Complete Genome Sequence of *Arthrobacter* Phage GantcherGoblin Exhibits Both Conservation with Subcluster AU6 Phages and Genetic Novelty

**DOI:** 10.1128/mra.00771-22

**Published:** 2022-11-02

**Authors:** J. J. Wheeler, Carina M. Carlos, Helen A. Cedzidlo, Xingfeiyang Liu, Massimo S. Modica, Izaiah D. Rhodes, Leah F. Truskinovsky, Ethan M. VanGosen, Hannah E. Gavin

**Affiliations:** a Tufts University, Medford, Massachusetts, USA; b Experimental College, Tufts University, Medford, Massachusetts, USA; c Department of Biology, Tufts University, Medford, Massachusetts, USA; DOE Joint Genome Institute

## Abstract

GantcherGoblin is a lytic siphovirus that was isolated on Arthrobacter globiformis B-2979 from soil collected in Massachusetts. The 55,368-bp genome has a GC content of 50.1% and 91 predicted protein-coding genes. Based on gene content similarity to phages in the Actinobacteriophage Database, GantcherGoblin was assigned to phage subcluster AU6.

## ANNOUNCEMENT

Bacteriophages (phages) are abundant and diverse members of the biosphere (1). Generating a comprehensive catalog of phages increases our chances of successfully applying these entities to medical and environmental challenges (2). Here, we report the discovery and characterization of the novel phage GantcherGoblin.

**FIG 1:**
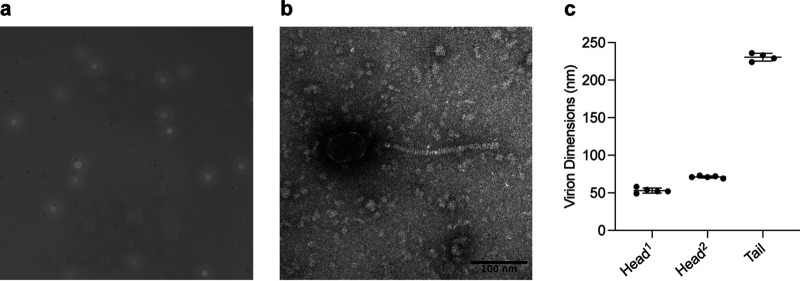
Characteristics of GantcherGoblin plaques and virions. (a) When plated on the isolation host Arthrobacter globiformis B-297 and incubated at 30°C, GantcherGoblin plaques exhibit clear centers and turbid margins. (b) Virions negatively stained with 1% uranyl acetate for TEM reveal siphoviral morphology, with prolate icosahedral heads and long tails. Scale bar = 100 nm (21, 22). (c) The dimensions of 5 virion capsids and 4 virion tails, measured using ImageJ, are plotted with a horizontal line at the calculated mean and error bars indicating the standard deviations of the mean (22). On average, GantcherGoblin tails measure 231 nm ± 5 nm. Capsids measure 53 nm ± 3 nm along the axis perpendicular to the tail (Head^1^) and 71 nm ± 1 nm along the same axis as the tail (Head^2^).

GantcherGoblin was isolated by undergraduate students at Tufts University Experimental College in partnership with the national Science Education Alliance-Phage Hunters Advancing Genomics and Evolutionary Science (SEA-PHAGES) program, using standard procedures (3, 4). Briefly, GantcherGoblin was extracted from a soil sample that had been collected in Medford, Massachusetts (42.410N, 71.115596W), in early September 2021, by washing the soil in peptone-yeast extract-calcium (PYCa) liquid medium. The wash was filtered (0.02-μm pore size), and a filtrate sample was plated in PYCa top agar with Arthrobacter globiformis B-2979. GantcherGoblin was purified through three consecutive rounds of plating with incubations at 30°C. Negative-staining transmission electron microscopy (TEM) revealed GantcherGoblin to exhibit siphoviral morphology (Fig. 1).

DNA was extracted from a plate-derived high-titer lysate using the Wizard DNA clean-up system (A7280; Promega) and submitted to the University of Pittsburgh, where a library was prepared for sequencing with a NEBNext Ultra II FS kit. The library was sequenced using an Illumina MiSeq system (v3 reagents), yielding 565,517 unpaired 150-bp reads (coverage, ~1,443×). Raw reads were assembled with Newbler v2.9 and checked using Consed v29 (5, 6).

Coding regions were initially identified by using DNA Master v5.0.2 to run GeneMark v4.9, Glimmer v3.02b, and ARAGORN v1.1 (7–9). Putative open reading frames (ORFs) and start positions were confirmed or revised through examination of Shine-Dalgarno sequences, Starterator reports, and BLAST searches against the PhagesDB Actinobacteriophage Database and NCBI BLASTn standard nucleotide databases (7, 10–12). Functions were assigned to ORFs by BLASTp searches against standard databases and HHpred searches against the following databases: PDB mmCIF70_12_Oct, Pfam-A v35, NCBI Conserved Domains (CD) v3.18, and PRD v6.9. Phamerator and the Comprehensive Antibiotic Resistance Database (CARD) v3.2.0 Resistance Gene Identifier (RGI) v5.2.1 were also consulted when predicting gene functions (13–15). When a function could not be assigned, TMHMM v2.0 and SOSUI were employed to identify transmembrane domains (16–18).

The GantcherGoblin genome is 55,368 bp, with a GC content of 50.1%. The genome termini have 9-bp 3′ single-stranded overhangs (5′-CGCCGGCCT-3′). All of the 91 protein-coding ORFs of GantcherGoblin are transcribed in the same direction. Of these genes, 87 have homologs in the Actinobacteriophage Database as of 31 May 2022. Functions were predicted for 26 GantcherGoblin genes; 11 encode virion structure or assembly components, while 15 encode nonstructural proteins. An additional 5 ORFs are predicted transmembrane proteins. No antibiotic resistance genes or tRNAs were identified. The absence of lysogeny-associated genes in the genome is consistent with GantcherGoblin plaque morphology and suggests an obligately lytic replication cycle (Fig. 1a).

Based on gene content similarity (GCS) to phages in the Actinobacteriophage Database as of 3 October 2022, GantcherGoblin was assigned to cluster AU, subcluster AU6 (12, 19, 20). GantcherGoblin shares 83.1% and 84.2% GCS with the two other subcluster AU6 phages, i.e., Uzumaki and Zeina, respectively. Like those of Zeina, the virions of GantcherGoblin have a prolate capsid structure (Fig. 1b and c).

Unusually large intergenic regions of up to ~200 bp are interspersed among Gantcher Goblin genes 32 to 36. In addition, the genomic region from bp ~13,000 to 16,000, which includes predicted minor tail proteins, has low conservation with other AU6 phages. The GantcherGoblin genome lacks several genes that are present in both Uzumaki and Zeina and, conversely, possesses novel genes (genes 48, 51, 76, and 87) with currently unknown functions.

### Data availability.

The complete GantcherGoblin genome is available in GenBank under accession number ON970564. GantcherGoblin raw sequencing data are archived under SRA accession number SRX14443506.
